# Nomograms for predicting overall survival and cancer-specific survival in elderly patients with epithelial ovarian cancer

**DOI:** 10.1186/s13048-023-01144-y

**Published:** 2023-04-14

**Authors:** Hao Cheng, Jin-Hong Xu, Xiao-Hong Kang, Chen-Chen Wu, Xiao-Nan Tang, Mei-Ling Chen, Zhu-Sheng Lian, Ning Li, Xue-Lian Xu

**Affiliations:** 1 Department of Radiation Oncology, The First Affiliated Hospital of Xinxiang Medical University, 88 Jiankang Road, Xinxiang, 453100 Henan China; 2 Department of Otolaryngology, AnYang District Hospital, Anyang, Henan China

**Keywords:** Dynamic nomogram, Epithelial ovarian cancer, Prognosis, Elderly, Risk stratification analysis

## Abstract

**Background:**

Epithelial ovarian cancer (EOC) is one of the most fatal gynecological malignancies among elderly patients. We aim to construct two nomograms to predict the overall survival (OS) and cancer-specific survival (CSS) in elderly EOC patients.

**Methods:**

Elderly patients with EOC between 2000 and 2019 were selected from the Surveillance, Epidemiology, and End Results (SEER) database. Enrolled patients were randomly divided into the training and validation set at a ratio of 2:1. The OS and CSS were recognized as endpoint times. The independent prognostic factors from the multivariate analysis were used to establish nomograms for predicting the 3-, 5- and 10-year OS and CSS of elderly EOC patients. The improvement of predictive ability and clinical benefits were evaluated by consistency index (C-index), receiver operating characteristic (ROC), calibration curve, decision curve (DCA), net reclassification improvement (NRI), and integrated discrimination improvement (IDI). Finally, the treatment efficacy of surgery and chemotherapy in low-, medium-, and high-risk groups were displayed by Kaplan–Meier curves.

**Results:**

Five thousand five hundred eighty-eight elderly EOC patients were obtained and randomly assigned to the training set (*n* = 3724) and validation set (*n* = 1864). The independent prognostic factors were utilized to construct nomograms for OS and CSS. Dynamic nomograms were also developed. The C-index of the OS nomogram and CSS nomogram were 0.713 and 0.729 in the training cohort. In the validation cohort, the C-index of the OS nomogram and CSS nomogram were 0.751 and 0.702. The calibration curve demonstrated good concordance between the predicted survival rates and actual observations. Moreover, the NRI, IDI, and DCA curves determined the outperformance of the nomogram compared with the AJCC stage system. Besides, local tumor resection had a higher benefit on the prognosis in all patients. Chemotherapy had a better prognosis in the high-risk groups, but not for the medium- risk and low-risk groups.

**Conclusions:**

We developed and validated nomograms for predicting OS and CSS in elderly EOC patients to help gynecologists to develop an appropriate individualized therapeutic schedule.

## Introduction

Ovarian cancer (OC) is one of the most lethal gynecological malignancies among women worldwide, with 81,584 new cases and 54,220 deaths in 2022 [1, 2]. Epithelial Ovarian cancer (EOC) is the most predominant pathologic subtype, accounting for over 90% of OC cases, which is most commonly diagnosed among women of post-menopausal age [3]. Although advanced medical techniques and drugs had been applied, the five-year survival rate of EOC is still below 50% [4, 5]. Usually, the treatment options for EOC patients mainly depend on the International Federation of Gynecology and Obstetrics (FIGO) stage as well as the American Joint Committee on Cancer (AJCC) stage. Recently, the prevalence of elder epithelial ovarian cancer is increasing as the population ages, which will increase the public health burden in the future [1, 2, 6–8]. However, unlike young patients with EOC, due to the worse physical and psychological conditions, the choice of treatment options in elderly patients has always been conservative, which usually relies on the experience of clinicians. Until now, there are no clear recommendations for elderly patients with EOC on treatment options. Consequently, to make better clinical decisions and accurately assess survival rate for clinicians, and benefit elderly EOC patients, it is imperative to define the survival factors and construct survival prediction models.

Nomogram is a widely used tool, which is shown as a comprehensive and readable model to predict the prognosis of patients diagnosed with cancer [9–11]. Compared with the FIGO stage and AJCC stage system, the nomogram model can predict the survival rate for each patient individually. At present, although several nomograms had been constructed for EOC patients in younger [12], under postoperative [13], and with site-distant metastases [14], there are no nomograms for the elderly EOC patient currently. Elderly patients were often attached to comorbidity, immunosenescence, and organ dysfunction, which lead to more treatment-related toxicity and poor prognosis for elderly patients [15]. Therefore, developing nomograms, especially for elderly EOC patients can improve the accuracy and actual value.

In this article, based on publicly available data from the Surveillance, Epidemiology, and End Results (SEER) database, we established and validated nomograms of overall survival (OS) and cancer-specific survival (CSS) in elderly EOC cases based on significant prognostic factors. Additionally, the C-statistic, calibration diagrams, and DCA curves were used to evaluate the discrimination and clinic utility of the nomograms. This study aimed to provide personalized survival predictions and optimize the clinical management of elderly EOC patients.

## Materials and methods

### Materials and variables

**Fig. 1:**
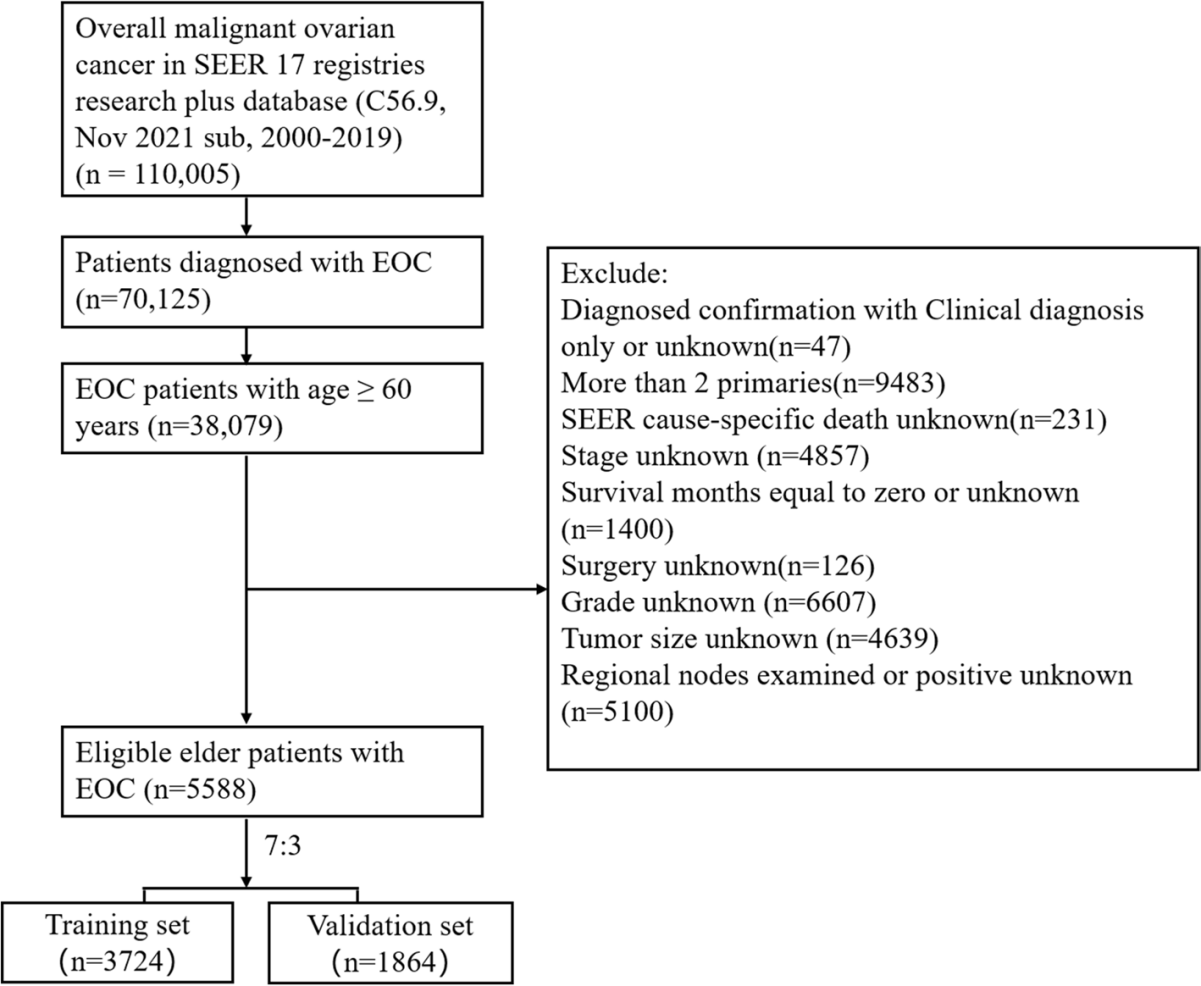
Flow diagram of the elderly epithelial ovarian cancer patients based on the inclusion and exclusion criteria

Data for this research was acquired from the Surveillance, Epidemiology, and End Results (SEER) Program—SEER research plus data (consisting of 17 populations based on cancer registration from 2000 to 2019) by utilizing the SEER*Stat software (SEER*Stat version 8.4.0.1). The medical ethics review and informed consent were not needed in this research. Cases were collected in terms of the inclusion criteria and exclusion criteria as follows. The inclusion criteria including: (1) Malignant epithelial ovarian cancer (EOC) cases (primary site recode of C56.9; ICD-O-3 codes including: serous (8441–8442,8460–8463,9014), mucinous (8144,8434,8470–8472,8480–8482), endometrioid/adenocarcinoma (8380–8383, 8560, 8570), clear cell (8310, 8313/3,8443–8444,9110), and others containing transitional cell as well as epithelial-stroma (8020–8021,8120,8122,8130, 8810,8890,8950,9000)). (2) Age ≥ 60 at diagnosis in patients with EOC. (3) pathologically confirmed; (4) only one primary malignant tumor; (5) active follow-up. The exclusion criteria: (1) SEER cause-specific death unknown (2) SEER historic stage A or AJCC stage unknown; (3) survival months equal to zero or unknown; (4) surgery unknown; (5) grade unknown, tumor size unknown and regional nodes examined or positive unknown. The workflow of this research was shown in Fig. 1.

We randomly divided all patients into training and validation groups by SPSS 20.0 at a ratio of 2:1. Nomogram was developed mainly based on the training set, while the evaluation and validation were performed by using the validation set. A total of twenty-two clinicopathological variables of EOC patients were extracted from the SEER database. The overall survival (OS) and cancer-specific survival (CSS) were considered endpoint times.

### Statistical analysis

R software (version 4.1.0) and SPSS 20.0 were utilized for all statistical analyses in this study. The baseline characteristics comparison between the training and validation group was performed by using the Chi-square test and Fisher’s exact test. Univariate Cox regression analysis was used to determine the association between clinicopathological variables and prognosis. Then, the multivariate Cox regression analysis was performed to find the independent prognostic factors for elderly patients with EOC (*P* < 0.05). Finally, the nomogram was established according to these independent prognostic variables.

The concordance index (C-index), the receiver operating characteristic (ROC), the calibration curve, the decision curve (DCA), the net reclassification improvement (NRI), and integrated discrimination improvement (IDI) were all calculated by R software. Among them, C-index, ROC, and the calibration curve were used to appraise the discriminative ability and the prediction efficiency of the nomogram. In addition, DCA, NRI as well as IDI were adopted to assess the predictive ability and effectiveness of the nomogram in comparison with the traditional AJCC stage system. New risk stratification was developed by X-tile software according to the total points of the nomogram, and patients were then separated into low, medium, and high-risk groups. The survival differences in different risk stratification groups were compared by log-rank test and Kaplan–Meier plots.

## Results

### Patient clinicopathologic characteristics

A total of 5,588 elderly EOC patients were finally included in this study according to the inclusion and exclusion criteria, which was randomly assigned to the training set (*n* = 3724) and validation set (*n* = 1864) at a ratio of 2:1 (Fig. 1). The median follow-up of these patients was 57 months (1–239 months). The clinicopathologic features of patients are shown in Table 1. The median age of all patients was 68 years old (range from 60 to 97). The majority patients were white (88.4%). Elderly EOC patients were often serous histologic type (68.5%), Grade III (47.2%), AJCC Stage III (46.5%), T3 (46.4%), N0 (51.3%), M0 (69.7%) and unilateral (62.4%). Most elderly EOC patients underwent local surgical resection (49.6%), debulking (46.1%), and chemotherapy (76.7%). The metastasis rates of liver and lung were significantly higher than those of bone and brain. Besides, significant difference in histology was found between the training and validation group.

**Table 1:** Characteristics of elderly patients with EOC in the training and validation groups

Characteristics	Total N (%)	Training group N (%)	Validation group N (%)	*P*
**Total**	5588	3724	1864	
**Age at diagnosis (median, years)**	68(60–97)	68(60–95)	68(60–97)	0.450
**Year of diagnosis (years)**				0.380
2000–2006	1672 (29.9%)	1117 (30.0%)	555 (29.8%)	
2007–2013	2802 (50.1%)	1847 (49.6%)	955 (51.2%)	
2014–2019	1114 (19.9%)	760 (20.4%)	354 (19.0%)	
**Race**				0.077
White	4940 (88.4%)	3315 (89.0%)	1625 (87.2%)	
Black	239 (4.3%)	145 (3.9%)	94 (5.0%)	
Other^a^	409 (7.3%)	264 (7.1%)	145 (7.8%)	
**Grade**				0.786
G1	431 (7.7%)	284 (7.6%)	147 (7.9%)	
G2	1015 (18.2%)	675 (18.1%)	340 (18.2%)	
G3	2635 (47.2%)	1773 (47.6%)	862 (46.2%)	
G4	1507 (27.0%)	992 (26.6%)	515 (34.2%)	
**Histology**				**0.003**
Serous	3825 (68.5%)	2590 (69.5%)	1235 (66.3%)	
Mucinous	276 (4.9%)	168 (4.5%)	108 (5.8%)	
Endo/adeno	900 (16.1%)	584 (15.7%)	316 (17.0%)	
Clear cell	392 (7.0%)	270 (7.3%)	122 (6.5%)	
Other^b^	195 (3.5%)	112 (3.0%)	83 (4.5%)	
**AJCC Stage**				0.385
I	1387 (24.8%)	939 (25.2%)	448 (24.0%)	
II	674 (12.1%)	443 (11.9%)	231 (12.4%)	
III	2597 (46.5%)	1707 (45.8%)	890 (47.7%)	
IV	930 (16.6%)	635 (17.1%)	295 (15.8%)	
**SEER Stage**				0.489
Local	1205 (21.6%)	819 (22.0%)	386 (20.7%)	
Regional	790 (14.1%)	518 (13.9%)	272 (14.6%)	
Distant	3593 (64.3%)	2387 (64.1%)	1206 (64.7%)	
**CA-125 Pretreatment**				0.941
Negative	305 (5.5%)	206 (5.5%)	99 (5.3%)	
Positive	2024 (36.2%)	1349 (36.2%)	675 (36.2%)	
Unknown	3529 (58.3%)	2169 (58.2%)	1090 (58.5%)	
**Laterality**				0.317
Unilateral	3486 (62.4%)	2317 (62.2%)	1169 (62.7%)	
Paired	49 (0.9%)	28 (0.8%)	21 (1.1%)	
Bilateral	2053 (36.7%)	1379 (37.0%)	674 (36.2%)	
**T Stage**				0.223
T1	1290 (23.1%)	868 (23.3%)	422 (22.6%)	
T2	748 (13.4%)	499 (13.4%)	249 (13.4%)	
T3	2592 (46.4%)	1746 (46.9%)	846 (45.4%)	
TX	958 (17.1%)	611 (16.4%)	347 (18.6%)	
**Surgery**				0.920
Pelvic exenteration	223 (4.0%)	152 (4.1%)	71 (3.8%)	
Debulking	2575 (46.1%)	1721 (46.2%)	854 (45.8%)	
Local resection	2774 (49.6%)	1841 (49.4%)	933 (50.1%)	
No surgery	16 (0.3%)	10 (0.3%)	6 (0.3%)	
**Lymph node examined** **(mean ± std)**	12.80 ± 12.119	12.96 ± 12.339	12.49 ± 11.664	0.192
**Lymph node positive** **(mean ± std)**	1.810 ± 4.853	1.830 ± 4.888	1.760 ± 4.781	0.655
**Radiation**				0.347
No /unknown	5525 (98.9%)	3678 (98.8%)	1847 (99.1%)	
Yes	63 (1.1%)	46 (1.2%)	17 (0.9%)	
**Chemotherapy**				0.075
No /unknown	1300 (23.3%)	893 (24.0%)	407 (21.8%)	
Yes	4288 (76.7%)	2831 (76.0%)	1457 (78.2%)	
**Bone metastasis**				0.675
No	2804 (50.2%)	1865 (50.1%)	939 (50.4%)	
Yes	6 (0.1%)	5 (0.1%)	1 (0.1%)	
Unknown	2778 (49.7%)	1854 (49.8%)	924 (49.6%)	
**Lung metastasis**				0.981
No	2728 (48.8%)	1815 (48.7%)	913 (49.0%)	
Yes	86 (1.5%)	57 (1.5%)	29 (1.6%)	
Unknown	2774 (49.6%)	1852 (49.7%)	922 (49.5%)	
**Liver metastasis**				0.995
No	2715 (48.6%)	1808 (48.5%)	907 (48.7%)	
Yes	97 (1.7%)	65 (1.7%)	32 (1.7%)	
Unknown	2776 (49.7%)	1851 (49.7%)	925 (49.6%)	
**Brain metastasis**				0.467
No	2806 (50.2%)	1867 (50.1%)	939 (50.4%)	
Yes	3 (0.1%)	3 (0.1%)	0(0.0%)	
Unknown	2779 (49.7%)	1854 (49.8%)	925 (49.6%)	

*Abbreviations: Endo/Adeno* endometrioid/adenocarcinoma, *AJCC* the seventh edition American Joint Committee on Cancer

^a^Other including American Indian/AK Native, Asian or Pacific Islander unknown

^b^Other including Adenocarcinoma with squamous metaplasia, Transitional cell carcinoma, Brenner tumor, malignant and NOS

### Independent prognostic factors in elderly patients with EOC

According to the result of univariate Cox regression analysis in the training set, a total of 19 variables were found to be associated with OS and CSS of elderly EOC patients, including age at diagnosis, year of diagnosis, race, grade, histology, laterality, lymph nodes examined, lymph nodes positive, AJCC stage, SEER stage, pretreatment CA125 level, T stage, chemotherapy, surgery, bone metastasis, liver metastasis, brain metastasis, and lung metastasis (*P* < 0.05). Independent prognostic factors related to OS and CSS were then decided by multivariate Cox analysis, 12 variables of age, race, grade, histology, laterality, AJCC stage, lymph nodes examined, lymph nodes positive, T stage, surgery, chemotherapy and bone metastasis were identified as OS-related these independent prognostic factors (*P* < 0.05), and 13 variables (age, race, grade, histology, laterality, AJCC stage, SEER stage, lymph nodes examined, lymph nodes positive, T stage, surgery, chemotherapy, and bone metastasis) were confirmed to be independent prognostic indicators for CSS. All these independent prognostic factors were included in the construction of the nomograms for predicting the OS and CSS of elderly patients with EOC. The detailed information was shown in Tables 2 and 3.

**Table 2:** Univariate and multivariate analyses of clinicopathologic parameters in elderly patients with EOC for predicting overall survival (OS)

Characteristics	Univariate analysis		Multivariate analysis	
	**HR (95%CI)**	***P***	**HR (95%CI)**	***P***
**Age at diagnosis (years)**	1.040(1.034–1.046)	** < 0.001**	1.040(1.034–1.046)	** < 0.001**
**Year of diagnosis (years)**				
2000–2006	Reference			
2007–2013	0.975(0.889–1.070)	0.597	0.990(0.868–1.128)	0.876
2014–2019	0.846(0.735–0.974)	**0.020**	0.942(0.776–1.143)	0.546
**Race**				
White	Reference		Reference	
Black	1.027(0.828–1.274)	0.809	1.161(0.934–1.444)	0.178
Other^a^	0.668(0.553–0.807)	** < 0.001**	0.744(0.615–0.900)	**0.002**
**Grade**				
G1	Reference		Reference	
G2	1.654(1.314–2.057)	** < 0.001**	1.285(1.023–1.615)	**0.031**
G3	2.634(2.143–3.237)	** < 0.001**	1.477(1.184–1.841)	**0.001**
G4	2.720(2.195–3.369)	** < 0.001**	1.430(1.135–1.802)	**0.002**
**Histology**				
Serous	Reference		Reference	
Mucinous	0.502(0.398–0.634)	** < 0.001**	1.257(0.973–1.624)	**0.080**
Endo/adeno	0.415(0.362–0.476)	** < 0.001**	0.851(0.731–0.991)	**0.038**
Clear cell	0.576(0.477–0.694)	** < 0.001**	1.489(1.217–1.823)	** < 0.001**
Other^b^	0.989(0.783–1248)	0.924	1.211 (0.955–1.535)	0.114
**AJCC Stage**				
I	Reference		Reference	
II	1.663(1.390–1.989)	** < 0.001**	1.652(1.262–2.162)	** < 0.001**
III	3.837(3.374–4.363)	** < 0.001**	2.451(1.987–3.024)	** < 0.001**
IV	5.274(4.556–6.104)	** < 0.001**	3.183(2.533–4.000)	** < 0.001**
**SEER Stage**				
Local	Reference		Reference	
Regional	1.843(1.548–2.195)	** < 0.001**	1.070(0.725–1.578)	0.734
Distant	3.892(3.411–4.441)	** < 0.001**	1.136(0.822–1.570)	0.440
**CA125 Pretreatment**				
Negative	Reference		Reference	
Positive	1.917(1.497–2.455)	** < 0.001**	1.072(0.833–1.380)	0.587
Unknown	1.849(1.451–2.357)	** < 0.001**	0.994(0.746–1.323)	0.965
**Laterality**				
Unilateral	Reference		Reference	
Paired	2.165(1.420–3.301)	** < 0.001**	1.116(0.727–1.713)	0.616
Bilateral	1.828(1.680–1.989)	** < 0.001**	1.257(1.146–1.378)	** < 0.001**
**T Stage**				
T1	Reference		Reference	
T2	1.765(1.478–2.107)	** < 0.001**	0.949(0.727–1.239)	0.698
T3	4.236(3.706–4.843)	** < 0.001**	1.401(1.132–1.735)	**0.002**
TX	2.657(2.271–3.109)	** < 0.001**	1.286(1.044–1.584)	**0.018**
**Surgery**				
Pelvic exenteration	Reference		Reference	
Debulking	0.915(0.755–1.110)	0.367	1.374(0.692–2.728)	0.364
Local resection	0.425(0.350–0.517)	** < 0.001**	0.738(0.600–0.907)	**0.004**
No surgery	1.676(0.850–3.304)	0.136	0.945(0.775–1.152)	0.575
**Lymph node examined**	0.990(0.986–0.993)	** < 0.001**	0.987(0.983–0.991)	** < 0.001**
**Lymph node positive**	1.032(1.026–1.037)	** < 0.001**	1.024(1.015–1.033)	** < 0.001**
**Radiation**				
No /unknown	Reference			
Yes	1.120(0.771–1.626)	0.552		
**Chemotherapy**				
No /unknown	Reference		Reference	
Yes	1.171(1.060–1.293)	**0.002**	0.801 (0.720–0.892)	** < 0.001**
**Bone metastasis**				
No	Reference		Reference	
Yes	5.026(2.085–12.114)	** < 0.001**	2.699(1.091–6.674)	**0.032**
Unknown	1.131(1.035–1.235)	**0.006**	1.117(1.014–1.230)	**0.024**
**Lung metastasis**				
No	Reference		Reference	
Yes	2.144(1.561–2.954)	** < 0.001**	1.245(0.891–1.738)	0.199
Unknown	1.153(1.055–1.261)	**0.002**	0.967(0.364–2.571)	0.946
**Liver metastasis**				
No	Reference		Reference	
Yes	1.699(1.237–2.334)	**0.001**	0.856(0.614–1.193)	0.358
Unknown	1.152(1.053–1.259)	**0.002**	2.226(0.807–6.145)	0.122
**Brain metastasis**				
No	Reference		Reference	
Yes	9.639(2.398–38.744)	**0.001**	4.387(1.083–17.771)	**0.038**
Unknown	1.128(1.033–1.232)	**0.007**	0.861(0.026–28.160)	0.933

*Abbreviations: Endo/Adeno* endometrioid/adenocarcinoma, *AJCC* the seventh edition American Joint Committee on Cancer

^a^Other including American Indian/AK Native, Asian or Pacific Islander unknown

^b^Other including Adenocarcinoma with squamous metaplasia, Transitional cell carcinoma, Brenner tumor, malignant and NOS

**Table 3:** Univariate and multivariate analyses of characteristics for predicting cancer specific survival (CSS) in elderly patients with EOC

Characteristics	Univariate analysis		Multivariate analysis	
	**HR (95%CI)**	***P***	**HR (95%CI)**	***P***
**Age at diagnosis (years)**	1.025(1.019–1.031)	** < 0.001**	1.025(1.018–1.032)	** < 0.001**
**Year of diagnosis (years)**				
2000–2006	Reference			
2007–2013	1.003(0.907–1.109)	** < 0.001**	0.993(0.860–1.146)	0.924
2014–2019	0.852(0.735–0.989)	** < 0.001**	0.923(0.751–1.135)	0.449
**Race**				
White	Reference		Reference	
Black	1.029(0.814–1.300)	0.812	1.203(0.950–1.523)	0.125
Other^a^	0.709(0.580–0.868)	**0.001**	0.807(0.659–0.988)	**0.038**
**Grade**				
G1	Reference		Reference	
G2	2.292(1.702–3.087)	** < 0.001**	1.653(1.221–2.237)	**0.001**
G3	4.210(3.186–5.564)	** < 0.001**	1.926(1.439–2.579)	** < 0.001**
G4	4.317(3.247–5.741)	** < 0.001**	1.819(1.347–2.455)	** < 0.001**
**Histology**				
Serous	Reference		Reference	
Mucinous	0.347(0.256–0.470)	** < 0.001**	1.211(0.874–1.678)	0.249
Endo/Adeno	0.280(0.235–0.334)	** < 0.001**	0.694(0.574–0.839)	** < 0.001**
Clear cell	0.561(0.458–0.687)	** < 0.001**	1.697(1.365–2.111)	** < 0.001**
Other	1.029(0.806–1.315)	0.816	1.287(1.003–1.650)	**0.047**
**AJCC Stage**				
I	Reference		Reference	
II	2.383(1.898–2.993)	** < 0.001**	1.654(1.007–2.715)	**0.047**
III	6.548(5.510–7.782)	** < 0.001**	2.559(1.685–3.884)	** < 0.001**
IV	9.274(7.697–11.173)	** < 0.001**	3.342(2.184–5.113)	** < 0.001**
**SEER Stage**				
Local	Reference		Reference	
Regional	2.932(2.332–3.686)	** < 0.001**	1.510(0.957–2.381)	0.077
Distant	7.279(6.042–8.769)	** < 0.001**	1.780(1.209–2.622)	**0.003**
**CA125 Pretreatmen**t				
Negative	Reference		Reference	
Positive	2.073(1.584–2.712)	** < 0.001**	1.029(0.783–1.354)	0.836
Other^b^	1.951(1.498–2.541)	** < 0.001**	0.970(0.711–1.324)	0.850
**Laterality**				
Unilateral	Reference		Reference	
Paired	2.508(1.627–3.865)	** < 0.001**	1.300(0.837–2.018)	0.243
Bilateral	2.033(1.855–2.227)	** < 0.001**	1.234(1.118–1.361)	** < 0.001**
**T Stage**				
T1	Reference		Reference	
T2	2.362(1.909–2.922)	** < 0.001**	0.939(0.685–1.288)	0.697
T3	6.346(5.367–7.502)	** < 0.001**	1.298(0.988–1.706)	0.061
TX	3.749(3.094–4.543)	** < 0.001**	1.256(0.959–1.646)	0.098
**Surgery**				
Pelvic exenteration	Reference		Reference	
Debulking	0.829(0.682–1.008)	**0.061**	0.947(0.414–2.167)	0.897
Local resection	0.336(0.275–0.410)	** < 0.001**	0.687(0.556–0.849)	**0.001**
No surgery	1.151(0.506–2.618)	0.737	0.908(0.743–1.111)	0.349
**Lymph node examined**	0.991(0.987–0.995)	** < 0.001**	0.988(0.984–0.992)	** < 0.001**
**Lymph node positive**	1.033(1.028–1.038)	** < 0.001**	1.021(1.012–1.030)	** < 0.001**
**Radiation**				
No /unknown	Reference			
Yes	1.203(0.817–1.772)	0.349		
**Chemotherapy**				
No /unknown	Reference		Reference	
Yes	1.398(1.247–1.568)	** < 0.001**	0.819(0.725–0.924)	**0.001**
**Bone metastasis**				
No	Reference		Reference	
Yes	5.442(2.257–13.121)	** < 0.001**	2.888(1.165–7.159)	**0.022**
Unknown	1.120(1.020–1.230)	**0.018**	1.134(1.023–1.257)	**0.016**
**Lung metastasis**				
No	Reference		Reference	
Yes	2.320(1.682–3.201)	** < 0.001**	1.209(0.861–1.697)	0.273
Unknown	1.145(1.042–1.259)	**0.005**	1.049(0.389–2.833)	0.924
**Liver metastasis**				
No	Reference		Reference	
Yes	1.836(1.331–2.533)	** < 0.001**	0.853(0.608–1.197)	0.357
Unknown	1.141(1.038–1.255)	**0.006**	1.699(0.565–5.111)	0.345
**Brain metastasis**				
No	Reference		Reference	
Yes	10.779(2.680–43.356)	**0.001**	4.595(1.133–18.638)	0.033
Unknown	1.117(1.017–1.226)	**0.020**	0.721(0.017–30.480)	0.864

*Abbreviations: Endo/Adeno* endometrioid/adenocarcinoma, *AJCC* the seventh edition American Joint Committee on Cancer

^a^Other including American Indian/AK Native, Asian or Pacific Islander unknown

^b^Other including Adenocarcinoma with squamous metaplasia, Transitional cell carcinoma, Brenner tumor, malignant and NOS

### Development and validation of the nomogram

**Fig. 2:**
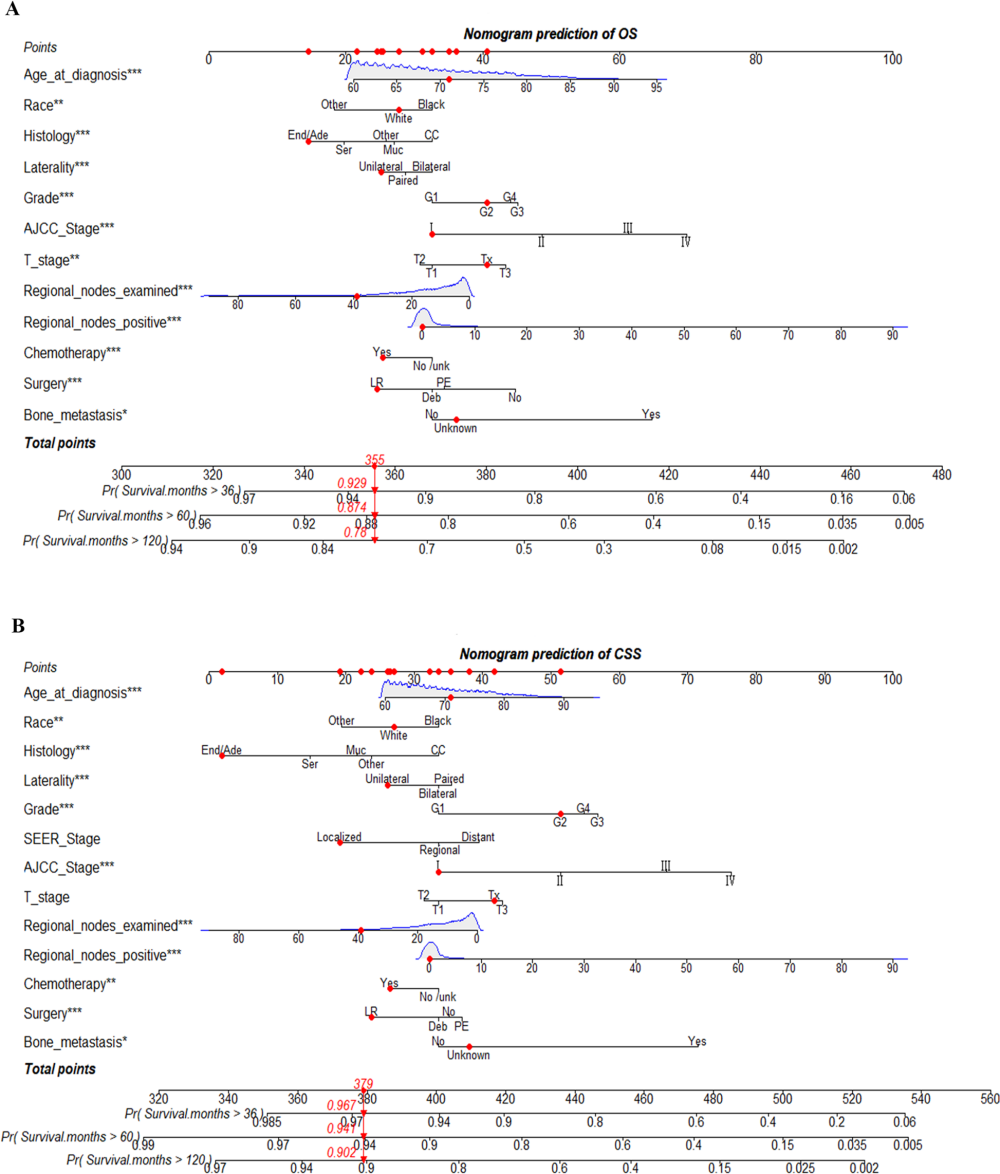
Nomograms to predict 3-, 5-, and 10-year overall survival and cancer-specific survival for elderly patients with epithelial ovarian cancer. *P < 0.05, **P < 0.01, ***P < 0.001

According to the identified independent prognostic factors, we constructed nomograms for elderly patients with EOC to predict 3-, 5-, and 10-year OS as well as CSS. The total score of all variables was calculated and then the 3-, 5-, and 10-year probability of OS and CSS could be concluded consequently. Figure 2A and B show the examples of using the nomogram to predict the overall survival probability and the cancer-specific survival probability of the given patient.

**Fig. 3:**
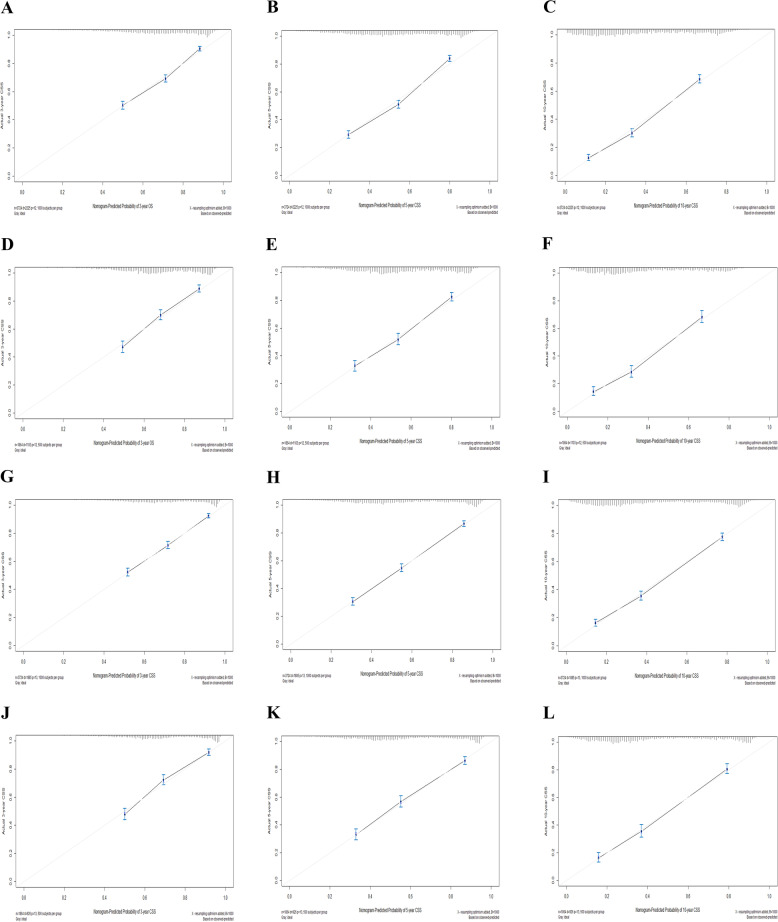
Calibration plots of 3-, 5-, and 10-year OS (**A**-**F**) and CSS (**G**-**L**) for elderly patients with epithelial ovarian cancer. **A**, **B**, **C** Calibration plots of 3-, 5-, and 10- year OS in the training cohort. **D**, **E**, **F** Calibration plots of 3-, 5-, and 10-year OS in the validation cohort. **G**, **H**, **I** Calibration plots of 3-, 5-, and 10-year CSS in the training cohort. **J**, **K**, **L** Calibration plots of 3-, 5-, and 10-year CSS in the validation cohort

C-index and calibration curve were carried out to assess the overall performance of the two nomograms. The C-index was 0.713 (95% CI: 0.701–0.725) in the training cohort and 0.751 (95% CI: 0.731–0.771) in the validation cohort for the prediction of OS, respectively. Moreover, the C-index for the prediction of CSS was 0.729 (95% CI: 0.717–0.741) and 0.702 (95% CI: 0.680–0.724) in the training and validation groups (Table 4). In addition, the calibration curves for the training and validation cohorts displayed a high consistency between the actual observed survival rates and those predicted by nomograms (Fig. 3).

**Table 4:** The NRI, IDI, and C-index of the nomograms and AJCC Stage system in OS and CSS prediction for elderly patients with EOC

Index	Training cohort		*P*	Validation cohort		*P*
	**Estimate**	**95%CI**		**Estimate**	**95%CI**	
**NRI (vs. AJCC Stage system)**						
For 3-year OS	0.220	0.173–0.250		0.207	0.147–0.279	
For 5-year OS	0.217	0.189–0.248		0.204	0.149–0.256	
For 10-year OS	0.163	0.125–0.230		0.165	0.087–0.261	
For 3-year CSS	0.230	0.194–0.259		0.192	0.154–0.269	
For 5-year CSS	0.265	0.227–0.315		0.204	0.150–0.276	
For 10-year CSS	0.247	0.180–0.298		0.190	0.135–0.294	
**IDI (vs. AJCC Stage system)**						
For 3-year OS	0.047	0.035–0.059	< 0.001	0.035	0.020–0.057	< 0.001
For 5-year OS	0.058	0.047–0.072	< 0.001	0.042	0.029–0.062	< 0.001
For 10-year OS	0.047	0.035–0.067	< 0.001	0.033	0.010–0.061	< 0.001
For 3-year CSS	0.041	0.031–0.055	< 0.001	0.036	0.024–0.062	< 0.001
For 5-year CSS	0.056	0.041–0.074	< 0.001	0.046	0.030–0.067	< 0.001
For 10-year CSS	0.053	0.037–0.075	< 0.001	0.043	0.025–0.074	< 0.001
**C-index**						
The nomogram (OS)	0.713	0.701–0.725		0.751	0.731–0.771	
The nomogram (CSS)	0.729	0.717–0.741		0.702	0.680–0.724	
The AJCC Stage (OS)	0.513	0.495–0.531		0.486	0.461–0.512	
The AJCC Stage (CSS)	0.492	0.476–0.508		0.500	0.477–0.524

**Fig. 4:**
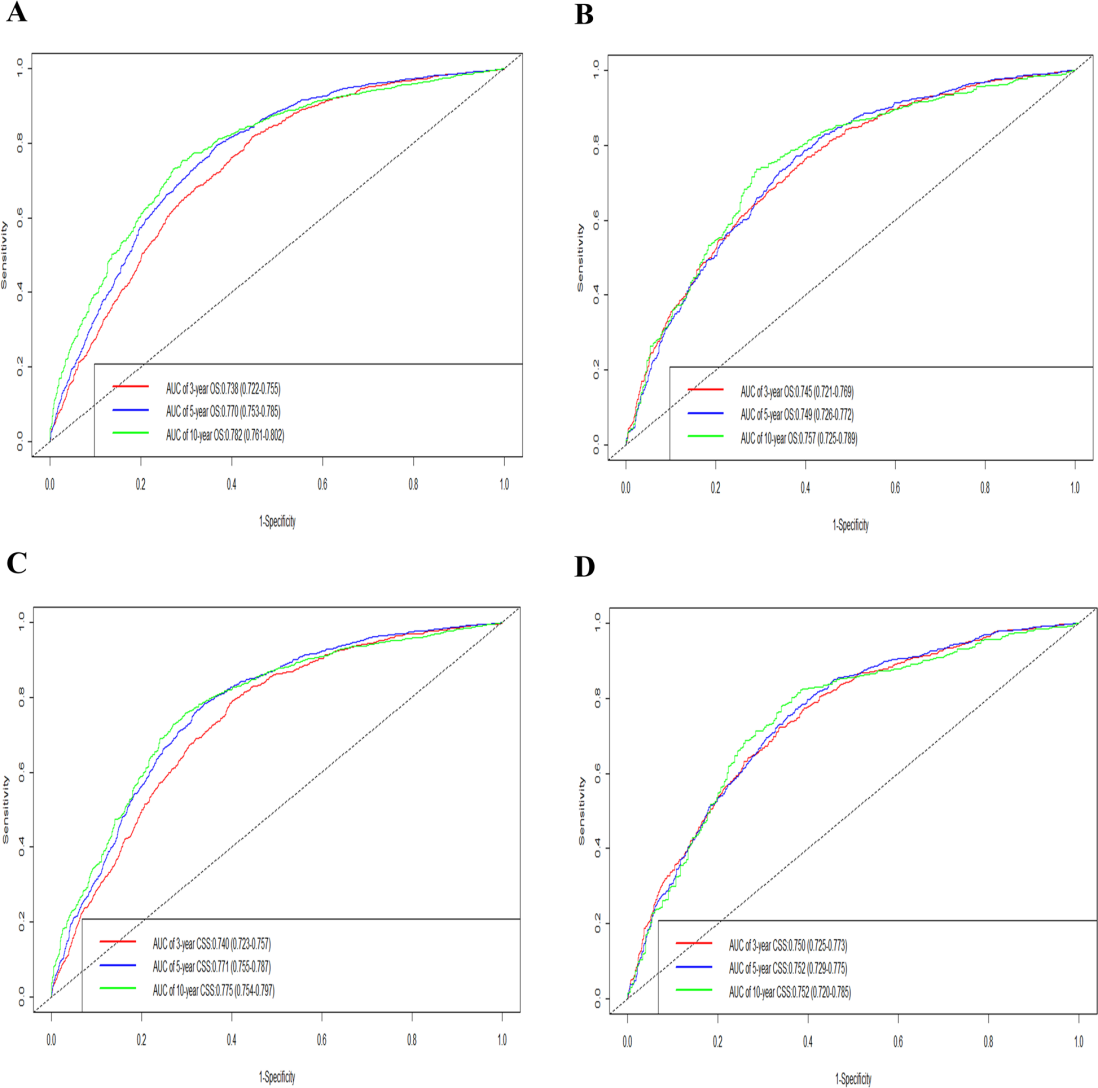
Time-dependent ROC curves of the nomogram for 3-, 5-, and 10-year predictions. AUC for predicting OS in the training set (**A**) and validation set (**C**); ROC curves corresponding to CSS in the training (**B**) and validation cohort (**D**), respectively

**Fig. 5:**
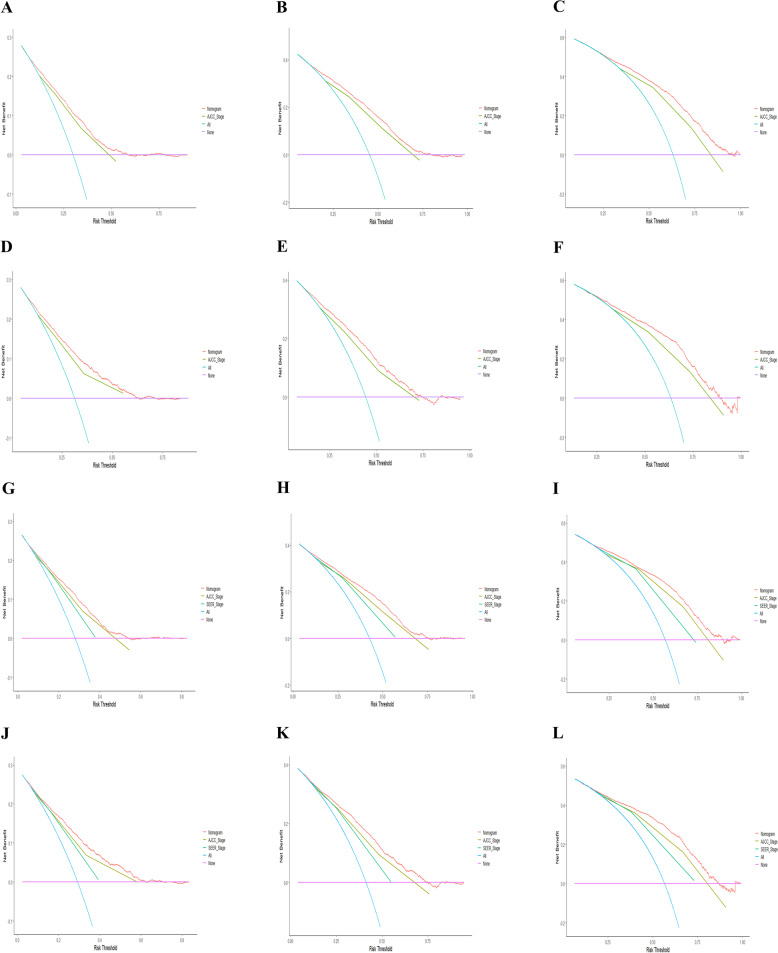
Decision curve analysis of the OS-associated and CSS-associated nomograms. DCA curves of 3-, 5-, and 10-year OS in the training cohort (**A, B, C**) and validation cohort (**D, E, F**). DCA curves of 3-, 5-, and 10-year CSS in the training group (**G, H, I**) and validation group (**J, K, L**)

The receiver operating characteristic curves (ROC) showed that the values of AUC at 3-, 5-, and 10-year for the prediction of OS were 0.738, 0.770, and 0.782 in the training group and 0.745, 0.749, and 0.757 in the validation group, respectively. Meanwhile, the 3-, 5-, 10-year AUC in the training cohort and validation cohort were 0.740, 0.771, 0.775 and 0.750, 0.752, 0.752 for the prediction of CSS (Fig. 4), which indicates that the nomograms had the good distinguishing ability. Additionally, the decision curves (DCA) in the training and validation group revealed good positive net benefits, which confirmed the superior prediction accuracy of the nomogram (Fig. 5).

### Clinical value comparison between nomograms and AJCC stage system

The application values of nomograms were estimated by NRI, IDI, and C-index in this study. The nomogram-related C-index were higher than that of the AJCC stage system (0.713 vs 0.513 in the training set, 0.751 vs 0.486 in the validation set for predictions of OS; meanwhile, 0.729 vs 0.492, 0.702 vs 0.500 in the training and validation group for predictions of CSS) (Table 4). The NRI for the 3-, 5-, and 10-year OS were 0.220 (95% CI: 0.173–0.250), 0.217 (95% CI: 0.189–0.248), and 0.163 (95% CI: 0.125–0.230), respectively. The IDI values for 3-, 5-, 10-year OS were 0.047 (95% CI: 0.035–0.059, *P* < 0.001), 0.058 (95% CI: 0.047–0.072, *P* < 0.001), and 0.047 (95% CI: 0.035–0.067, *P* < 0.001). These results were also certificated in the validation group (Table 4), which indicates that the nomogram had a preferable predictive capacity compared with the AJCC stage system. In addition, DCA curves also revealed that the nomogram had better prediction OS and CSS probability compared to the AJCC stage system.

### Risk stratification for elderly patients with EOC

**Fig. 6:**
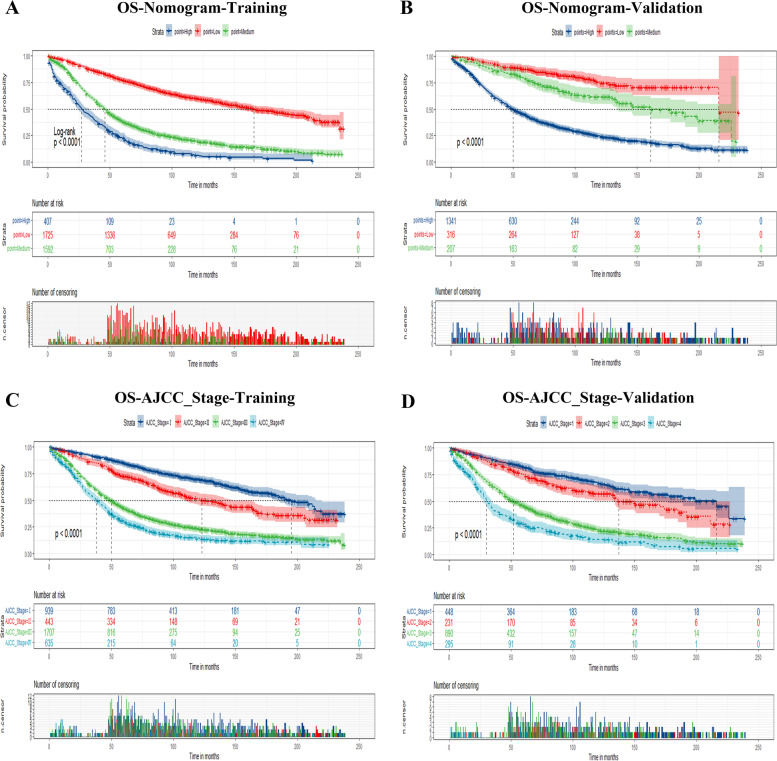
Kaplan–Meier curves of elderly patients with EOC for predicting OS. **A, B** Kaplan–Meier curves in the training (**A**) and validation cohorts (**B**) according to the new risk stratification system. **C, D** Kaplan–Meier curves according to the AJCC Stage system of the training (**C**) and validation cohorts (**D**)

**Fig. 7:**
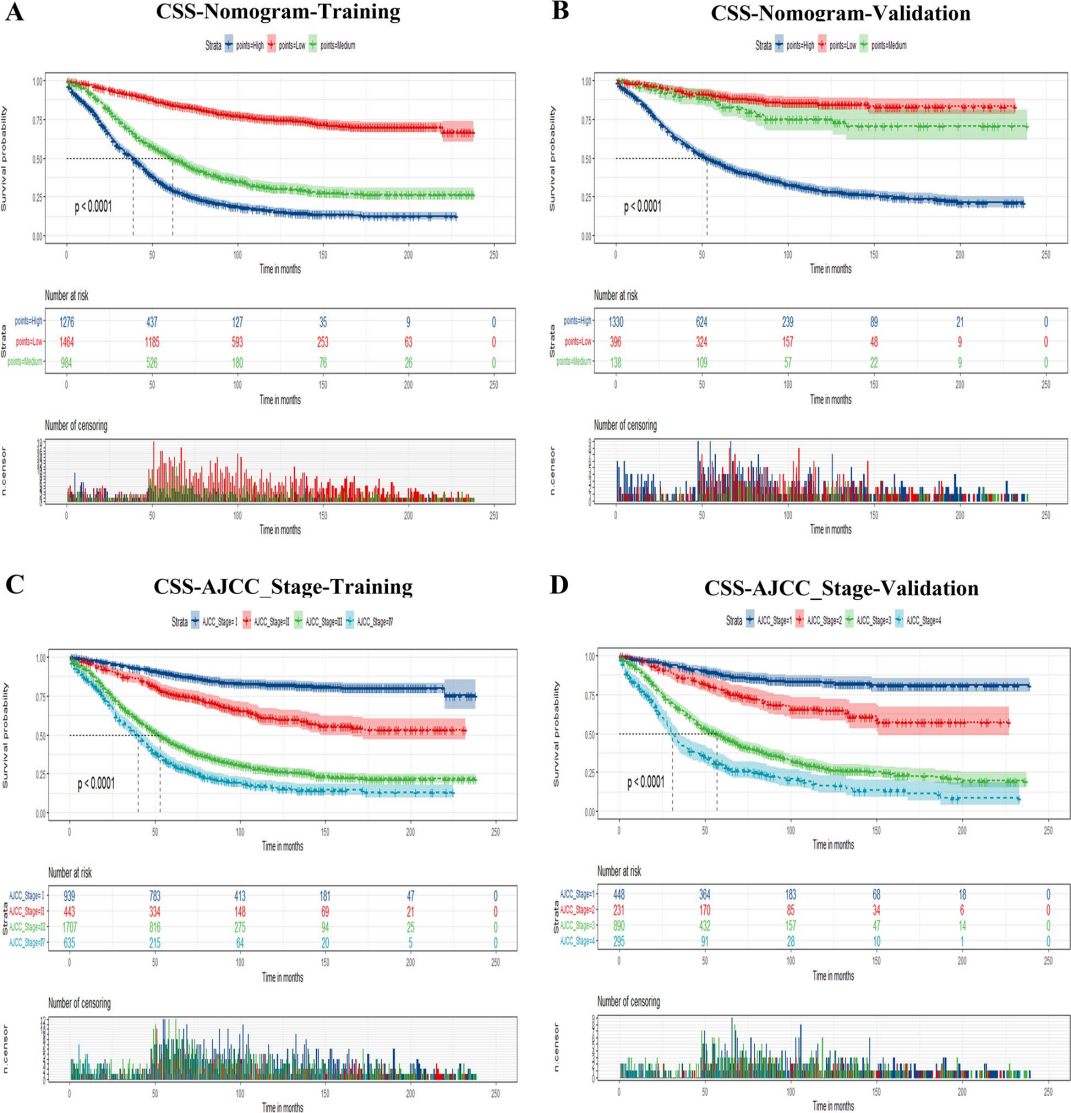
Kaplan–Meier CSS curves of elderly patients with EOC based on the new risk stratification system and the AJCC stage system. **A, B** Kaplan–Meier CSS curves based on the new risk stratification system in the training (**A**) and validation cohorts (**B**). **C, D** Kaplan–Meier CSS curves according to the AJCC stage system in the training (**C**) and validation cohorts (**D**)

Furthermore, total scores were calculated according to the nomogram for risk stratification. Elderly patients with EOC were therefore classified into three risk groups for the prediction of OS, including low-risk (total points ≤ 140.91), medium-risk (140.94 ≤ total points ≤ 177.33), and high-risk (total points ≥ 177.35). Kaplan–Meier curves showed a statistically discriminatory for all three subgroups in both training and validation groups, whereas the AJCC stage system had shown inadequate ability to distinguish mortality risk, especially in stage III and stage IV, similarly in stage I and stage II (Figs. 6 and 7).

**Fig. 8:**
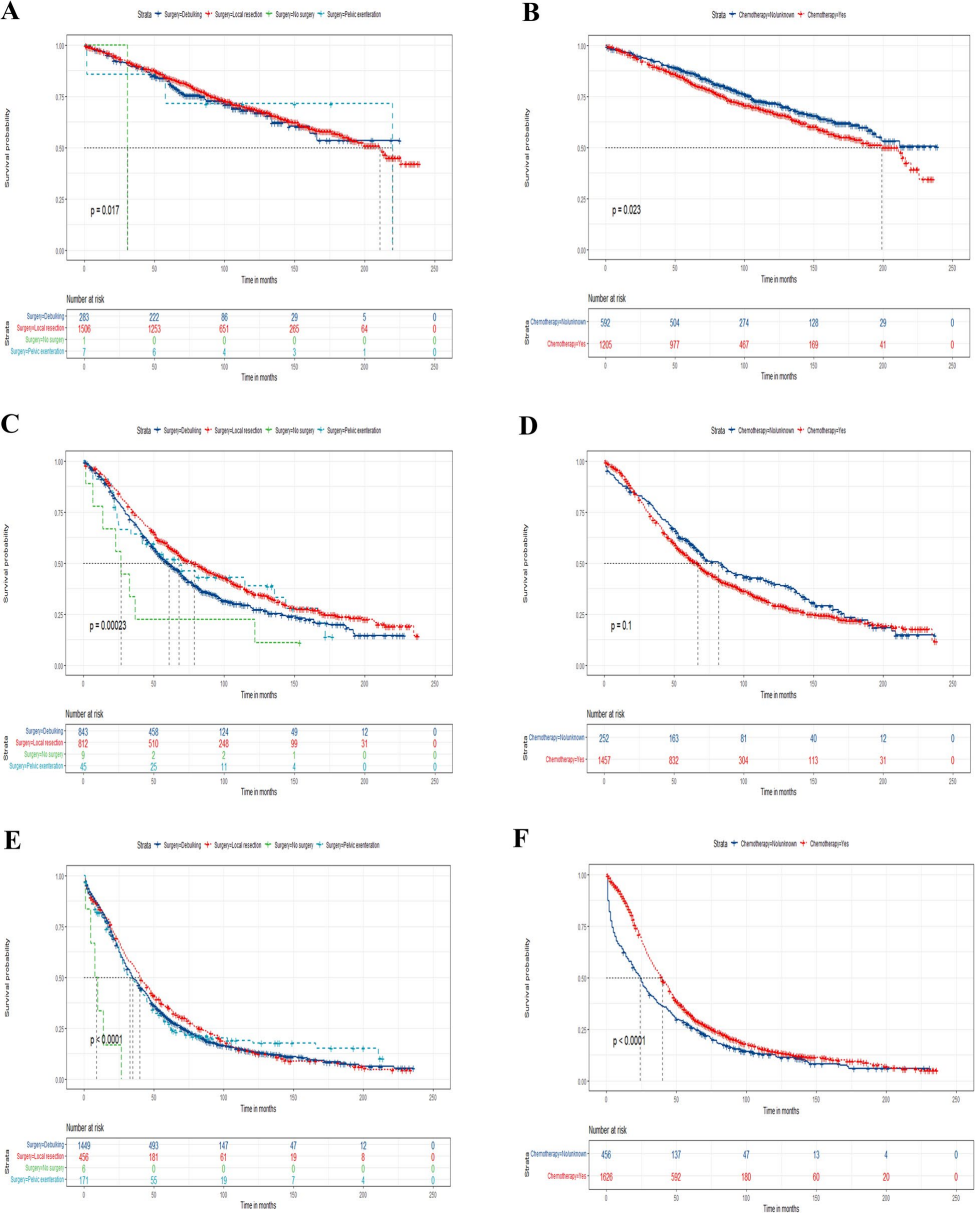
Kaplan–Meier curves for predicting OS based on the new risk stratification system of all elderly EOC patients. Kaplan–Meier OS curves of patients with various surgery types in the low- (**A**), medium-(**C**), and high-risk group (**E**); Kaplan–Meier curves of patients with or without chemotherapy in the low- (**B**), medium-(**D**), and high-risk group (**F**) for predicting OS

**Fig. 9:**
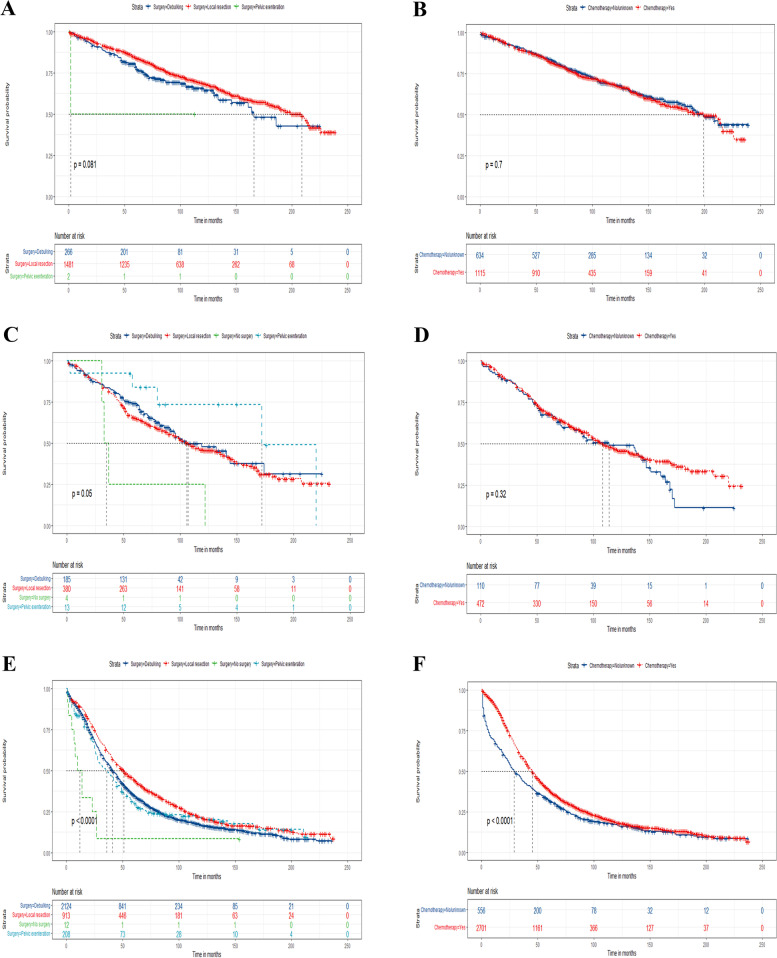
Kaplan–Meier CSS curves for all elderly EOC patients based on the new risk stratification system. Kaplan–Meier CSS curves of patients with various surgery types in the low- (**A**), medium- (**C**), and high-risk group (**E**); Kaplan–Meier curves of patients with or without chemotherapy in the low- (**B**), medium- (**D**), and high-risk group (**F**) for predicting CSS

In addition, surgery and chemotherapy were both independent prognostic factors in elderly patients with EOC, we attempt to clarify the efficacy of treatment including surgery and chemotherapy in the above three risk stratification subgroups by log-rank test and Kaplan–Meier curves. According to our results, elderly patients with EOC undergoing local tumor resection had a higher survival rate compared with debulking as well as pelvic exenteration (Fig. 8). Finally, it was obvious that elderly patients with EOC receiving chemotherapy had a better prognosis in the high-risk groups, but for the medium-risk and low-risk groups, chemotherapy was incapable of improving the outcome (Fig. 9).

### Development of dynamic web-based calculators for these nomograms

Based on the model, two dynamic web-based calculators were constructed to simplify the application of these nomograms, which can be accessible via https://xxlchxjh.shinyapps.io/DynNomapp/ for OS and https://xxlchxjh.shinyapps.io/DynNomappCSS/ for CSS. Survival probability can obtained by the online calculator conveniently.

## Discussion

Epithelial ovarian cancer is one of the most common and fatal gynecologic tumors, which is often diagnosed in postmenopausal women. Recently, researchers mainly focused their studies on patients with EOC at childbearing age [12, 16, 17]. Studies on elderly patients with EOC were extremely rare relatively. In addition, with the change in demographic structure, there would be more elderly patients diagnosed with EOC or other tumors in the future, and the burden of cancer among the elderly will become even heavier with the aging of the population in the world [18, 19]. Due to a variety of factors, there were wide differences between younger and elderly patients with EOC in the prognosis and treatment decisions. However, there was no prognostic model specifically for elderly patients with EOC until now. The clinical management was a new challenge for gynecologists in elderly patients with EOC. Therefore, a specialized prognostic model for elderly patients with EOC should be developed and more attention should be paid.

Previously, various factors had been found to be related to the prognosis of patients with EOC, such as race, grade, histology, tumor size, lymph nodes examined, T stage, N stage, FIGO stage, AJCC stage, surgery, chemotherapy, serum CA125 level, surgery, age, residual lesion size, liver metastasis, bone metastasis, and lung metastasis. Therefore, we included as many of these factors as possible in our study. By univariate and multivariate Cox regression analysis, we found that a total of 12 variables were significantly related to OS, and 13 variables were meaningfully related to CSS, which were included in the two nomograms, respectively. According to the results of our study, advanced age was positively associated with a worse prognosis in EOC patients. There were several factors that might contribute to the poorer prognosis of EOC patients with age, including worse nutritional status, performance status, more complex underlying diseases as well as poor tolerance to treatment. Taylor Jolyn S et al. found that Non-white elderly women are less likely to receive the standard of care treatment for ovarian cancer and more likely to die from their disease than white elderly women [20]. The lipophilic statin used after surgery which might provide cardio-protection can improve the overall survival in elderly patients with epithelial ovarian cancer in the study of Vogel Tilley Jenkins et al. [21]. Larissa A Meyer et al. found that neoadjuvant chemotherapy was failing to improve the prognosis of patients at age 80 or greater in EOC patients with stage III, but with decreased perioperative morbidity [22]. Additionally, older patients tend to be more immune senescence, which might lead to the worse effect of immunotherapy [23, 24].

At present, the efficacy of surgery, chemotherapy, and radiotherapy were still unclear for elderly patients with EOC. Whether or not to choose surgery, chemotherapy, and radiotherapy for these patients had aroused many disputes in gynecologists as no consistent guidance [25, 26]. In our study, it was obvious that radiotherapy makes no contributions to improving the prognosis of elderly patients with EOC, while, surgery and chemotherapy can significantly improve the outcome of these patients in comparison. Nevertheless, it is commonly that not every elderly patient with EOC can get benefits from surgery or chemotherapy. According to the new risk stratification system, we found that patients who underwent local tumor resection had higher survival rates than other surgery types in the medium and high-risk group. Moreover, the efficacy of chemotherapy also appeared different in these three risk groups, it was obvious that patients who accept chemotherapy were more likely to have better outcome in the high-risk groups, but for the medium and low-risk group, chemotherapy could not contribute to the increase survival rate. So, elderly patients with EOC in the low-risk group should accept local resection without chemotherapy, while, for elderly patients with EOC in the high-risk group, chemotherapy with local resection is the better choice.

Based on the SEER database and the independent prognostic factors in our study, we established and validated two nomograms for predicting the prognosis of elderly patients with EOC in this study. Results of the validation of these nomograms indicated that they had an excellent predictive and discriminative performance. We established two new risk stratification systems for elderly patients with EOC by calculating the total score for each patient based on the two nomograms, which also showed a good ability to differentiate risk groups compared with the AJCC stage system.

This study also had some defects indisputably. Firstly, variables’ information like specific chemotherapy regimens, residual tumor size, concomitant underlying disease conditions, surgical complications, and side effects of chemotherapy were unavailable from the SEER database. Secondly, some correlated prognostic variables like residual tumor size and surgical complication were not contained in our nomograms which resulted in some limitations in our studies. Finally, there is inevitable selection bias as this study is based on a retrospective database, external validation should be performed to provide more reliable evidence.

## Conclusion

There were two prognostic nomograms and risk stratification systems constructed in this study based on the public SEER database. These two nomograms and risk stratification systems show good predictive efficacy and excellent clinical benefit, which can be used to predict survival and help gynecologists to develop a more appropriate individualized therapeutic schedule for each elderly patient with EOC.

## Data Availability

The data of this study is available for all authors.
